# Integrated Finite Element Modeling and High-Speed Impact Synthesis: A Novel Pathway for Embedded Al/W Energetic Composites

**DOI:** 10.3390/ma19153252

**Published:** 2026-08-01

**Authors:** Kunkun Song, Xin Yu, Yi Lan, Xiansheng Wang, Gang Liu

**Affiliations:** High Speed Aerodynamics Institute, China Aerodynamics Research and Development Center, Mianyang 621000, China; songkk2025@163.com (K.S.); xinyu20260625@163.com (X.Y.); 17723445672@163.com (Y.L.)

**Keywords:** finite element simulation, high-speed impact, impact velocities, embedded microstructure, Al/W energetic composites

## Abstract

**Highlights:**

- An integrated simulation-experimental approach for the accelerated design of novel energetic materials.
- FEM analysis revealed stress distributions and energy conversion during heterogeneous particle impacts.
- FEM-guided synthesis of embedded Al/W composites by simulating high-speed impact processes.
- Al/W energetic composites exhibited 3.5 times enhanced reaction enthalpy (8806.0 ± 152 J/g) versus pure Al.

**Abstract:**

Propelled by accelerated technological innovation, the advancement of new material development has become imperative for emerging industries. The finite element method, leveraging multi-scale modeling and interdisciplinary integration, has established itself as one of the pivotal computational tools for significantly boosting research and development efficiency in material science. This study employed a combined approach of ABAQUS simulation and experimentation to efficiently design and synthesize an Al/W energetic composite characterized by concentrated energy release, high reaction enthalpy, and a unique discrete embedded structure. The finite element analysis focused on the stress distribution, equivalent strain, embedding depth, and energy conversion phenomena of heterogeneous particles under varying impact velocities. The results demonstrated that at a collision velocity of 500 m/s, embedded collisions between Al and W particles were achieved, providing effective guidance for synthesizing Al/W energetic composites with embedded structural features via high-speed impacts. TG-DSC analysis revealed that the Al/W energetic composites exhibited a reaction enthalpy change of 8806.0 ± 152 J/g and a maximum heat flow rate of 101.5 ± 2.8 W/g, which were 3.5-fold and 3.1-fold higher than those of pure Al with identical dimensions, respectively, and significantly surpassed the values of the mechanically mixed Al/W energetic composites. This integrated finite element simulation and experimentation provides a new pathway towards the efficient design and synthesis of novel materials with analogous components and structures.

## 1. Introduction

In the development of modern composite materials, numerical simulation technology has evolved from a supplementary tool into a core driver throughout the entire process of material design, parameter optimization, and performance prediction [1,2,3]. By establishing high-fidelity mathematical models to replace or substantially reduce physical experiments, this approach systematically predicts critical behaviors of materials, such as stress distribution and deformation trends, in multi-physics-coupled environments. Consequently, it validates the rationality of material processing parameters, thereby mitigating research and development (R&D) costs and engineering risks while providing essential data support for structural improvements in novel materials [4,5]. The finite element method (FEM) is indispensable across engineering industries, structural design, scientific research, and interdisciplinary fields, owing to its robust capacity for handling complex systems, high computational efficiency, cost-effectiveness, and superior multi-physics coupling capabilities [6,7]. In particular, the integration of artificial intelligence and quantum computing is poised to further enhance the computational efficiency and design precision of finite element simulations, thereby driving paradigm shifts in industrial and scientific research. This evolution will transform FEM into a “virtual laboratory” for material innovation [8,9].

Composite materials, as novel materials characterized by exceptional performance and design flexibility, not only depend on chemical composition but are also significantly governed by their microstructure [10]. However, the correlation between microscopic structures and macroscopic properties is highly complex. Finite element meso-mechanical analysis provides a powerful means to elucidate this correlation, clarify the underlying mechanisms governing property variations, and thereby directly provide a theoretical guide for composite design [11]. As a representative example, ABAQUS has emerged as a core simulation tool in material design and R&D, owing to its comprehensive library of constitutive models, robust nonlinear analysis capabilities, multi-physics coupling functionalities, and flexible secondary development interfaces. It is extensively applied in composite material fabrication, novel alloy development, and functional material innovation [12,13]. For instance, researchers utilized its finite element analysis to conduct biomimetic design of bamboo microstructures, effectively mitigating stress concentrations [14]. Furthermore, post-processing studies on material microstructural simulations via ABAQUS provide critical insights into micro-mechanical behaviors [15]. Thus, finite element simulations have become an indispensable tool for composite microstructure design, significantly enhancing performance prediction accuracy and accelerating R&D cycles [16].

In the field of energetic materials, two-phase flow fabrication technology effectively mitigates particle agglomeration issues associated with conventional mechanical mixing methods, providing a viable approach for the design and synthesis of Al-based energetic materials (Al-EMs) [17,18]. The core advantages of this technology lie in its enhanced mixing and mass transfer, induced mechanochemical reactions, and precise control over powder morphology and composite structures, thereby overcoming the limitations of traditional methods and significantly improving material performance [19,20]. Moreover, controllable preparation and development of composite microstructures require extensive experimental trial and error, which severely impedes R&D efficiency and application potential. Consequently, finite element simulations provide an efficient methodology for advancing novel material development and designing unique microstructures [21,22].

In this study, ABAQUS was employed to model the high-energy impact processes between two metal particles with significantly different Young’s modulus at ambient temperatures. The evolution patterns of the stress response, equivalent plastic strain, embedded depth, and energy conversion during heterogeneous particle impacts were systematically investigated. Based on the simulation results and a self-developed gas-solid two-phase flow material preparation platform, an Al/W energetic composite with an embedded microstructure was designed and synthesized. The simulation effectively realized the dynamic embedding behavior of heterogeneous particles and guided the optimization of composite preparation parameters, ultimately enabling the fabrication of composites with high energy-release characteristics and unique microstructures. This study demonstrated the notable advantages and potential of finite element simulations in advancing the synthesis of high-performance composites, providing effective guidance for developing metal/ceramic-based composites with analogous structures.

## 2. Finite Element Simulation of High-Speed Impact of Al-W Particles

**Figure 1:**
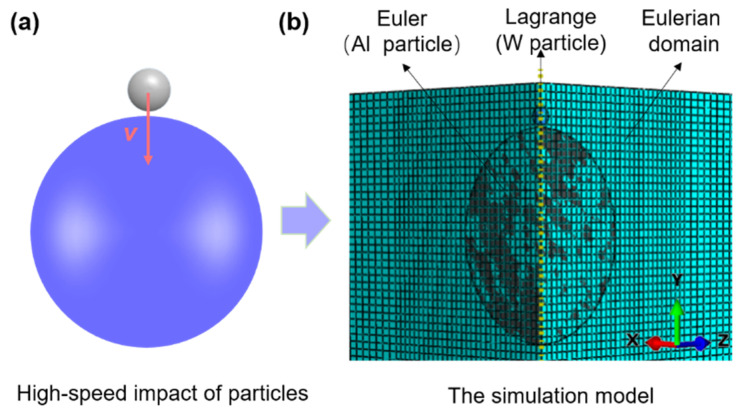
High-speed impact model for particulate materials. (**a**) Schematic illustration of high-speed impact between heterogeneous particles. (**b**) The coupled Eulerian–Lagrangian impact model.

The core process of composites preparation via gas–solid two-phase flow was selected as the simulation subject. This process primarily relies on the gas phase to drive heterogeneous particles toward high-speed impact (Figure 1a), enabling the embedded integration of the particles with significant differences in hardness, elastic modulus, and size. Therefore, the impact velocity, as one of the critical parameters, was intensively investigated. In the field of high-speed impacts, the Johnson–Cook (J-C) material model is widely adopted to predict contact behaviors, deformation mechanisms, and energy conversion under large deformations and high-speed impacts [23,24]. This model describes the plastic flow behavior of metallic materials at high strain rates or elevated temperatures by coupling strain hardening, strain rate sensitivity, and thermal softening effects [25,26]. The constitutive expression is given by Equation (1):

(1) $$\sigma_{y}=(A+B\varepsilon_{p}^{n})(1+C\mathrm{In}{\dot{\varepsilon }}^{*})(1-T^{*m})$$

Therein, $\sigma_{y}$ is the equivalent flow stress, and the parameter *A* denotes the initial yield stress under the reference condition. *B* and *n* represent the strain hardening modulus and its exponent, respectively, *C* was the strain rate hardening coefficient, and *m* was the thermal softening index. $\varepsilon_{p}$ represents the equivalent plastic strain; ${\dot{\varepsilon }}^{*}$ represents the dimensionless plastic strain rate; and $T^{*m}$ is the normalized temperature, which is defined as follows [27]:

(2) $$T^{*m}=\left\{\begin{array}{c} 0;\\ (T-T_{\mathrm{trans}})/(T_{\mathrm{melt}}-\\ 1; \end{array}\right.T_{\mathrm{trans}});\begin{array}{c} T<T_{\mathrm{trans}}\\ T_{\mathrm{trans}}\leq T\leq \\ T_{\mathrm{melt}}<T \end{array}T_{\mathrm{melt}}$$

where $T_{\mathrm{melt}}$ is the melting temperature above which the material is fluid and the hardening effect should totally vanish. $T_{\mathrm{trans}}$ is a reference transition temperature at or below which there is no temperature dependence of the response.

The J-C constitutive model is an empirical model that is widely applied to characterize the plastic behavior of metallic materials under conditions involving high strain rates, large deformations, and elevated temperatures. As the most extensively used constitutive equation in finite element simulations of particle impact [28,29], the coupled Eulerian–Lagrangian model was adopted for this study (Figure 1b).

In Al-EMs, the primary components consist of reactive aluminum (Al) powder and oxidizers. Typically, oxidizers comprise metals/non-metals, metal/non-metal oxides, organic/inorganic compounds, or polymers [30,31,32,33]. In this study, tungsten (W) particles, which have a high density, Young’s modulus, shear modulus, and melting point, were selected as the oxidizer. Al particles served as the matrix. The detailed material properties were shown in Table S1. Additionally, considering that the collision process between Al and W particles involved coupled thermo-mechanical behaviors and a rapidly evolving temperature field, the contact friction coefficient at the two-phase interface was set to 0.2, in accordance with Ref. [27]. Finite element analysis was performed using ABAQUS 2022 software with a coupled Eulerian–Lagrangian (CEL) model to investigate the preparation conditions for the impact embedding of micron-scale particles. The Eulerian domain employed the eight-node thermally coupled Eulerian hexahedral elements (EC3D8RT) while the Lagrangian mesh utilized the eight-node linear hexahedral elements (C3D8R), both utilizing a reduced integration with hourglass stabilization. The mesh was discretized based on a size criterion, and the element size was set to 0.001, which corresponded to 1/20 of the characteristic dimension of the Al matrix. To prevent material loss, zero normal velocity constraints were applied to all six boundary surfaces of the external Eulerian domain. Initial conditions comprised a uniform temperature field of 298 K, with Al particles maintained at static positions while W particles imparted impact velocities ranging from 100 m/s to 900 m/s at 200 m/s intervals to establish relative motion for impact-embedding phenomena. The heterogeneous particulate system (Al–W) featured a controlled size ratio of 2 μm:20 μm. Material constitutive models and meshing configurations were implemented, as illustrated in Figure 1b, incorporating Johnson–Cook plasticity criteria for both metallic phases. The velocity-dependent deformation mechanisms were systematically analyzed across the prescribed impact spectrum to identify critical embedding thresholds.

## 3. Materials and Methods

The Al powder (purity: 99.9%, particle size: ~20 μm) and W powder (purity: 99.9%, particle size: ~2 μm) were supplied by Wuhan Aoke New Material Technology Co., Ltd (Wuhan, China). Experiments were conducted using a self-designed gas-solid two-phase flow apparatus [34], which was modified to utilize the low-pressure (~1 MPa), low-velocity (~10 m/s), and low-temperature nitrogen gas (<0 °C) accelerated through an annular jet ejector. This configuration drove solid particles to undergo high-speed impact (~500 m/s) for 20 min, followed by spiral classification to synthesize the Al/W energetic composite powder.

High-resolution microstructural images were characterized by Scanning Electron Microscopy (SEM; Sigma 300, Zeiss, Oberkochen, Germany) and Transmission Electron Microscopy (TEM; Tecnai F20, FEI, Hillsboro, OR, USA), achieving resolutions of 1.0 nm and 2.4 nm, respectively. Both instruments were equipped with Energy Dispersive Spectrometers (EDSs) for elemental mapping analysis. The temperature-dependent mass variation and heat flow profiles of raw Al, W, and Al/W energetic composites were analyzed a via simultaneous Thermogravimetric Analysis and Differential Scanning Calorimetry (TG-DSC; STA 509 Jupiter Classic, NETZSCH, Selb, Germany). TG-DSC analyses were conducted on all samples under an air atmosphere across a temperature range of 25 °C to 1300 °C at a heating rate of 10 °C/min, with each measurement performed in triplicate to ensure reproducibility.

## 4. Results and Discussion

### 4.1. Numerical Simulation Analysis of High-Speed Impacts Between Al/W Particles

**Figure 2:**
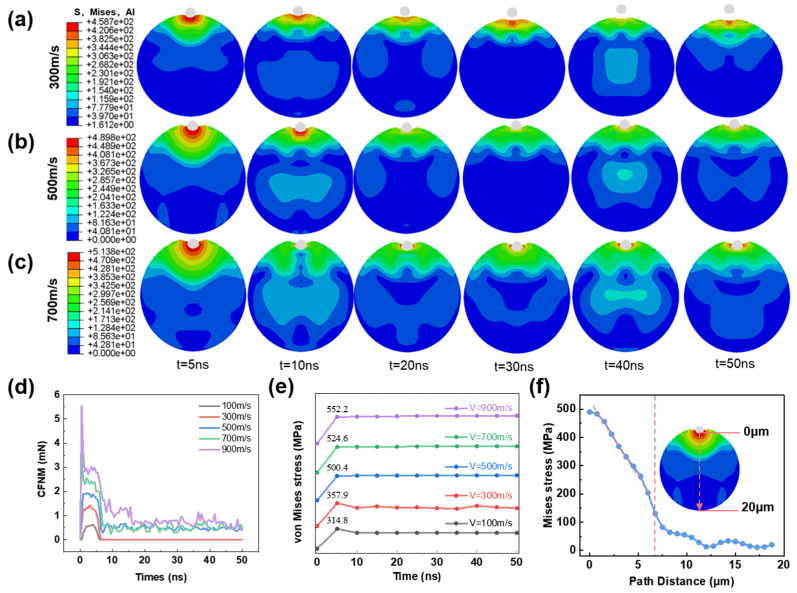
Mechanical response and stress analysis from numerical simulations. (**a**–**c**) Stress nephograms with time evolution at velocities of 300 m/s, 500 m/s, and 700 m/s. (**d**) Contact force normal magnitude (CFNM) response at the central point of the Al matrix. (**e**) Comparative analysis of von Mises stress evolution under multiple velocity conditions. (**f**) Gradient stress profile along the central axis interface of the Al matrix at 500 m/s and 5 ns.

The high-speed impacts of metallic particles exhibited extreme characteristics, including ultra-short durations, large strains, high strain rates, and rapid temperature transients [35,36]. As illustrated in the force and stress analyses during the collision process in Figure 2, at velocities below 900 m/s, the particles completed the processes of impact contact, rebound, or embedment within an extremely short timeframe (<50 ns). Specifically, at velocities ≤ 300 m/s, distinct rebound behaviors of W particles from Al particles were observed after contact (Figure 2a). Conversely, at velocities ≥ 500 m/s, W particles effectively embedded into the Al matrix, with the penetration depth increasing progressively with higher impact velocities (Figure 2b,c). Concurrently, high-speed impacts induced pronounced stress concentration in the Al matrix, exhibiting a gradient stress distribution. The contact force normal magnitude (CFNM) at the contact points of the Al matrix was extracted to characterize the mechanical response, thereby providing a direct visualization of stress evolution. At impact velocities of 100 m/s and 300 m/s, CFNM exhibited an initial rapid increase followed by a decline to 0 mN, indicating rebound behavior (Figure 2d). In contrast, at velocities ≥ 500 m/s, the peak CFNM increased with the rising impact velocity. Although a similar rapid increase and subsequent decrease were observed, CFNM stabilized above 0 mN after 20 ns. As shown in Figure 2e, the maximum stress within the Al matrix rose from 314.8 MPa to 552.2 MPa as the impact velocity increased. Notably, the internal stress persisted over time without dissipation. This metastable feature facilitated enhanced internal energy in the Al–W system, suggesting its potential for superior energy release characteristics. On the other hand, the sensitivity of the maximum von Mises stress in the Al matrix to yield strengths, mesh sizes, and contact conditions (such as friction coefficients) was evaluated at a specific impact velocity (500 m/s). The results showed that, within a reasonable parameter range, the maximum peak stress always occurred at the Al–W contact interface, exhibiting similar stress contours (as shown in Figure S1). The maximum variation in peak stress was less than 10%, demonstrating good reliability of the simulation results. To perform an in-depth analysis of stress gradient variations in the Al matrix following particle impact, cross-sectional stress was extracted at a specific collision velocity (500 m/s). As evidenced in Figure 2f, the internal stress within the Al matrix exhibited a piecewise decrease with increasing distance from the contact point of the heterogeneous particle. Specifically, within 7 μm of the contact point, the stress decreased approximately linearly. Beyond this region, it declined gradually and remained relatively stable with minor fluctuations.

**Figure 3:**
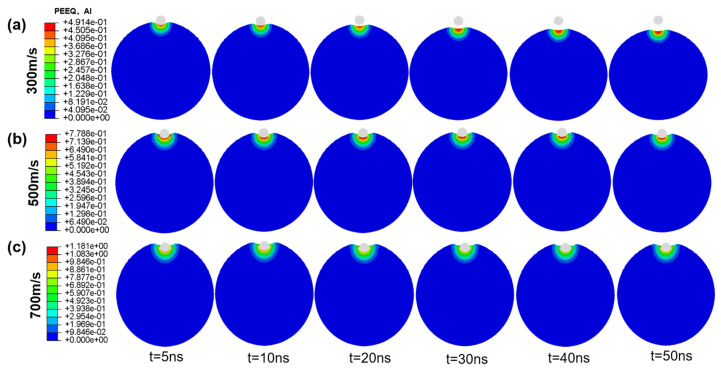
Plastic strain nephograms. (**a**–**c**) Plastic strain nephograms with time evolution at collision velocities of 300 m/s, 500 m/s, and 700 m/s.

Compared to W, Al metal exhibited a relatively low yield strength (≤100 MPa, significantly lower than von Mises stress of 314.8 MPa at 100 m/s collision velocity) and exceptional plastic strain capacity [37,38]. Therefore, during the finite element simulations, plastic strain was predominantly concentrated within the Al matrix. As illustrated in Figure 3, the maximum plastic strain values observed during impacts at 300 m/s, 500 m/s, and 700 m/s were primarily distributed in the Al matrix, reaching magnitudes of 0.49, 0.79, and 1.18, respectively. Similar to the stress nephograms, the separation occurred between the W particles and Al matrix after 20 ns of impact contact at collision velocities ≤ 300 m/s (Figure 3a). At velocities ≥ 500 m/s, however, the plastic deformation of the Al matrix intensified with increasing impact velocities (Figure 3b,c). Additionally, plastic deformation exhibited localized significant localization under all tested velocities. High-strain regions were concentrated within the contact zone and directly beneath it, with strain concentration forming at t = 5 ns. As collisions progressed (t = 10~20 ns), the high-strain zone expanded slightly inward along the impact direction. At the late stage (t = 30~50 ns), the strain distribution stabilized, confirming the irreversible plastic deformation and residual strain retention in the Al matrix. Moreover, the plastic strain nephograms indicated that the maximum values consistently occurred locally at the Al–W interface, where robust interfacial bonding was observed. These findings provided quantitative simulation data and experimental insights for understanding W particle embedding mechanisms and designing tightly bonded interfaces in composite particles.

**Figure 4:**
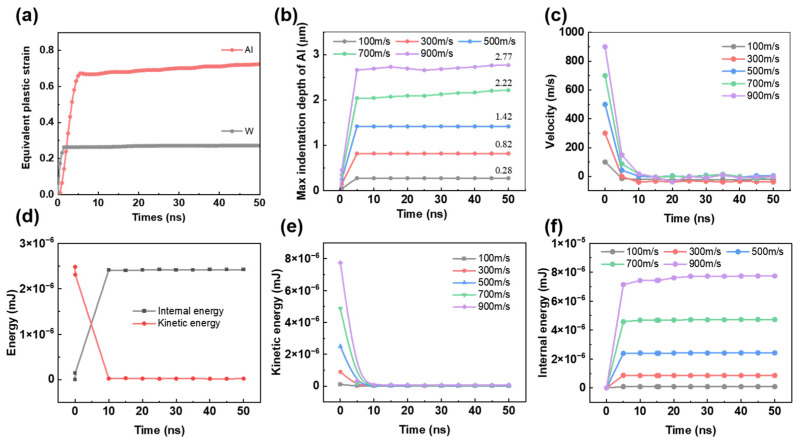
Numerical simulation analyses of plastic strain, embedded depth, velocity, and energy evolution. (**a**) Equivalent plastic strain of W particles and the Al matrix at 500 m/s. (**b**) Evolution of maximum indentation depth of the Al matrix over time. (**c**) Velocity variation over time. (**d**) Energy conversion process of composite particles during 500 m/s impacts. (**e**,**f**) Kinetic energy and internal energy variations under different impact velocities.

On the other hand, data on the equivalent plastic strain, depth of indentation, and energy evolution under different collision velocities were extracted to analyze the embedding behaviors and energy transformation mechanisms during high-speed impacts. As shown in Figure 4a, the time–equivalent plastic strain curves of the W particle and Al matrix at 500 m/s revealed that both particles reached their peak strains within an extremely short duration (<5 ns). The peak strain of the Al matrix was approximately 2.7 times higher than that of the W particles, indicating that plastic deformation of the Al matrix dominated the high-speed impact process. Furthermore, displacement analysis at the contact points demonstrated that the maximum indentation depth of Al matrix increased with rising collision velocity from 0.28 μm to 2.77 μm (Figure 4b). At low velocities (≤300 m/s), the W particles rebounded completely after impact. However, owing to the pronounced plastic deformation induced in the Al substrate during impact, a permanent crater remained on the Al surface, even after the particles had detached from the substrate and the contact force had returned to zero. On the other hand, analysis of the influences of yield strengths, mesh sizes, and contact friction coefficients on the maximum indentation depth of the Al matrix revealed that the higher the yield strength of the matrix phase (e.g., Al matrix) and the friction coefficient between the two phases, the smaller the indentation depth of W particles embedded into the matrix, with the variation constrained within ±7.7%. When the mesh size was set to 0.001, the maximum indentation depth of the Al matrix nearly converged (Figure S2), confirming that the simulation results were sufficiently reliable. Collisions between Al and W particles inevitably induced velocity variations and energy conversion. As illustrated in Figure 4c, higher collision velocities led to more pronounced velocity decay within an ultra-short duration (5 ns), with all curves eventually stabilizing until reaching 0 m/s. The velocity change directly reflected the kinetic energy variation in particles. Taking the 500 m/s collision case as an example (Figure 4d), the energy evolution trend exhibited continuous conversion of kinetic energy to internal energy, where the internal energy predominantly manifested as plastic deformation energy within the particles. The kinetic energy decay curves and internal energy growth curves under different collision velocities in Figure 4e,f further confirmed this conclusion. Specifically, during 0 to 50 ns at 500 m/s collision, the total kinetic energy decreased from 2.48 × 10^−6^ mJ to 2.32 × 10^−8^ mJ, while the total internal energy increased from 0 mJ to 2.42 × 10^−6^ mJ. The conversion rate from kinetic energy to internal energy reached approximately 97.6%, with the remaining 2.4% of energy dissipated in other forms (e.g., thermal energy dissipation induced by high-speed impacts). Consequently, high-speed impacts effectively enhanced the internal energy storage of Al/W energetic composites. Nevertheless, for pure Al energetic materials with a high energy density of approximately 31 kJ/g [39], the fraction of internal energy enhanced by high-speed impact conversion remained quite limited. For example, at a collision velocity of 500 m/s, the maximum kinetic energy converted per unit mass was merely 125 J/g, which constituted only a negligible portion relative to its stored chemical energy.

### 4.2. Preparation of Al/W Energetic Composites with Embedded Microstructures

**Figure 5:**
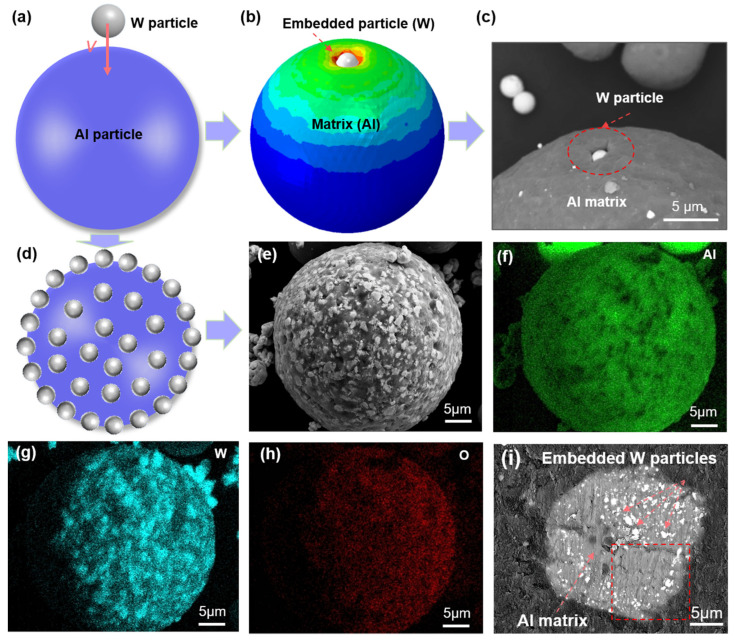
Design and synthesis of embedded Al/W energetic composites and their microstructural characteristics. (**a**–**c**) Simulation and physical morphologies of W particles embedded in the Al matrix. (**d**,**e**) Expected renderings and actual microstructural characteristics of embedded Al/W composite particles. (**f**–**h**) The distribution characteristics of Al, W, and O elements on the surface of Al/W composite particles. (**i**) Embedded microstructure within Al/W composite particles after metallographic polishing.

When the collision velocity was too low (≤300 m/s), the heterogeneous particles (W) caused only minor deformation on the Al matrix surface, failing to achieve the effective embedment into the matrix or the formation of composite particles with an embedded microstructure. Conversely, excessive collision velocities induced severe deformations of the Al matrix, especially under repeated cyclic impacts, which significantly compromised their sphericity. Therefore, to fabricate Al-based energetic composites with an embedded configuration, a collision velocity range of 500~700 m/s was identified as optimal based on the simulation results. Accordingly, a gas-solid two-phase flow apparatus was employed to drive high-hardness, small-sized W particles (~2 μm) and Al particles (~20 μm) via inert gas (N_2_) at approximately 500 m/s, aiming to synthesize Al/W energetic composites with embedded microstructures. Figure 5a–c presented the simulation and experimental results of a single W particle embedded in an Al matrix at a collision velocity of ~500 m/s. After 20 min of cyclic impacts, Al/W composite particles with discretely embedded microstructural features were successfully fabricated. The schematic illustration and experimental micrographs of multiple W particles embedded in the Al matrix were displayed in Figure 5d and Figure 5e, respectively.

EDS analysis confirmed the homogeneous distribution of Al, W, and O elements on the composite surface, with no significant interfacial defects observed (Figure 5f,h). High-resolution SEM imaging, bright-field TEM imaging, and elemental mapping collectively confirmed this characteristic (Figure S3). SEM images, after metallographic polishing, clearly visualized the internal embedded microstructures of Al/W composite particles, wherein W particles were discretely distributed within the Al matrix (Figure 5i). Statistical analysis of the W particles’ embedded depth over one quarter of the region shown in Figure 5i yielded an average value of 1.725 μm (Table S2), which was 21.48% higher than the simulated embedding depth (1.42 μm). This discrepancy likely originated from the fact that the high-speed impact fabrication process involved particle swarm collisions, in which W particles underwent multiple impacts with the Al matrix, possibly increasing the actual embedding depth. The observed deviation fell entirely within the acceptable range. Furthermore, the comprehensive microstructural characteristics of Al/W composites subjected to impact velocities of 300 m/s, 700 m/s, and 900 m/s further confirmed that an adjacent impact velocity of ~500 m/s constituted the optimal condition for fabricating Al/W composites exhibiting satisfactory sphericity of the Al matrix and a favorable embedding effect (as depicted in Figure S4). Thus, these results collectively validated the effectiveness and feasibility of the approach combining simulation and experimental design. This preparation method was analogous to the cold spray principle. In both processes, powder particles were accelerated by a high-speed gas stream and underwent solid-state deposition onto a substrate [40]. The entire procedure was conducted at a relatively low temperature, which minimized the oxidation loss and activity degradation of Al during fabrication (persistent challenges in mechanical alloying and spark plasma sintering), and thereby fundamentally preserved the safety and reactivity of the energetic composite [41,42]. Moreover, this approach overcame the deficiency of weak interfacial bonding between particles commonly encountered in cold spray methods [43].

### 4.3. Energy Release Characteristics of Al/W Energetic Composites

**Figure 6:**
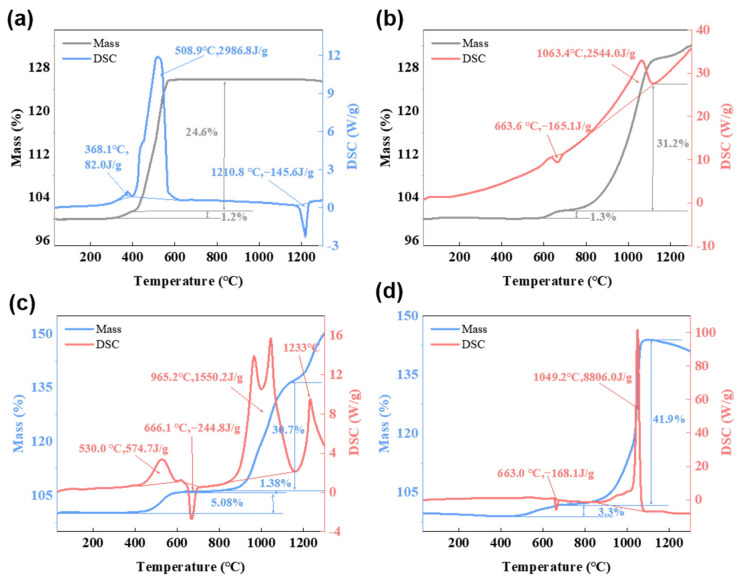
Thermal behavior characterization of raw materials and Al/W energetic composites. (**a**,**b**) TG-DSC curves of Al and W raw materials. (**c**) TG-DSC curve of mechanically mixed Al/W energetic composites with identical compositions. (**d**) TG-DSC curve of embedded Al/W composites.

TG-DSC analysis was employed to further investigate the energy release characteristics of Al/W energetic composites with embedded microstructures in comparison to those prepared by mechanical mixing. This comparison further validated the superior energy release performance of the embedded microstructures synthesized via high-speed impacts. Specifically, the TG-DSC curves of raw materials revealed two distinct exothermic peaks for pure W within the range of 30 °C to 1250 °C, occurring at 386.1 °C and 508.9 °C. These peaks represented the gradual oxidation of W atoms, with corresponding reaction enthalpies of 82.0 J/g and 2986.8 J/g. Furthermore, an endothermic peak attributed to WO3 vaporization was observed at approximately 1210.8 °C, with a reaction enthalpy change of −145.6 J/g (Figure 6a). Pure Al exhibited a dominant exothermic peak at approximately 1063.4 °C, with an enthalpy change of 2544.0 ± 34 J/g and a mass increase of 31.2%, indicating an intense reaction process of Al atoms. The maximum heat flow rate during energy release reached 32.9 ± 1.2 W/g. Additionally, an endothermic peak appeared at around 663.6 °C, with an enthalpy change of −165.1 J/g (Figure 6b). Notably, no mass variation was detected during this process, confirming a phase transition of molten Al (melting point of Al: 660 °C) [44]. The Al/W energetic composite (19.4 wt.% W) prepared by mechanical mixing (10 min) displayed three distinct exothermic peaks at approximately 530.0 °C, 965.2 °C, and 1233.0 °C. Among these, the primary exothermic peak comprised two overlapping sub-peaks, with a total enthalpy change of 1550.2 ± 7.6 J/g and a maximum heat flow rate of 15.7 ± 0.9 W/g. This pronounced reduction was predominantly attributed to inferior interfacial contact and severe agglomeration of the reinforcement particles (W), which were inherent limitations of the mechanical mixing process. Within this mixture, the restricted interaction at the Al matrix–oxygen interface and the high propensity of W particles, acting as heat sinks, to agglomerate synergistically impeded the exothermic oxidation, as well as the self-sustaining propagation of the combustion reaction. Consequently, this compromised the overall exothermic performance of the Al matrix in an oxygen atmosphere. In contrast, the Al/W energetic composites synthesized via impact velocity-designed gas-solid two-phase flow exhibited an endothermic peak at approximately 663.0 °C due to the Al melting, accompanied by a sharp, high-intensity exothermic peak at 1049.2 °C in DSC. This indicated highly concentrated exothermic behavior, with a reaction enthalpy change of 8806.0 ± 152 J/g and a maximum heat release rate of 101.5 ± 2.8 W/g. These values were 3.5 times and 3.1 times higher than those of pure Al. Correspondingly, oxygen atom absorption during oxidation induced a 41.9% mass increase, exceeding pure Al by 10.7%. Various thermal performance parameters exceeded those of mechanically mixed Al/W energetic composites. The substantial increase in reaction enthalpy is attributed to the embedded W particles acting as thermal conductors or catalysts, which promote more complete oxidation of the Al matrix during in-air testing. Consequently, the embedded structure synthesized by the high-velocity impact fabrication method effectively enhances the combustion efficiency of Al-based energetic composites. The entire fabrication process was simple and efficient, attributed to the precise optimization of high-speed impact parameters via finite element simulations, and provided a novel alternative microstructural engineering strategy for synthesizing advanced composites with analogous structures.

## 5. Conclusions

Accelerating the development of novel materials has emerged as a critical demand for emerging industries. FEM, leveraging its capabilities in multi-scale modeling and interdisciplinary integration, has become one of the core computational tools for enhancing research and development efficiency. Combining ABAQUS simulations with experimental validation, this study efficiently designed and synthesized Al/W energetic composites characterized by concentrated energy release, high reaction enthalpy change, and embedded microstructures. First, numerical analysis of the pivotal particle impact stage systematically revealed the stress response, equivalent strain, embedded depth, and kinetic-to-internal energy conversion mechanisms of micron-scale heterogeneous particles at varying collision velocities (100~900 m/s). A critical embedding threshold was identified at ~500 m/s, providing essential process parameters for synthesizing embedded Al/W energetic composites via gas–solid two-phase flow technology. Subsequently, SEM and EDS characterizations confirmed the formation of a typical embedded microstructure at a 500 m/s collision velocity. Finally, TG-DSC curves indicated that the embedded Al/W energetic composite exhibited a reaction enthalpy change of 8806.0 ± 152 J/g and a maximum exothermic heat flow rate of 101.5 ± 2.8 W/g, which were 3.5 times and 3.1 times higher than that of pure Al, respectively. Furthermore, the composite simultaneously surpassed the mechanically mixed Al/W energetic counterparts with identical compositions, and this enhancement was primarily attributed to the embedded configuration induced by high-speed impact synthesis. Consequently, the systematic integration of finite element simulation and experimental validation provided a new pathway for the efficient design and synthesis of advanced materials with analogous components and structures. This methodology demonstrated significant engineering applicability for accelerating new material development and advancing emerging industries.

## Data Availability

The original contributions presented in this study are included in the article/Supplementary Materials. Further inquiries can be directed to the corresponding authors.

## Supplementary Materials

The following supporting information can be downloaded at: https://www.mdpi.com/article/10.3390/ma19153252/s1, Figure S1: Sensitivity analysis of the effects of mesh size, the key J-C model parameters, and contact conditions on stress contours. (a) Influence of mesh size. (b) Influence of yield strength. (c) Influence of friction coefficient; Figure S2: Sensitivity of the maximum indentation depth in the Al matrix to variations in mesh size, the key parameters of the J-C model, and contact conditions. (a) Sensitivity of the maximum indentation depth to mesh size. (b) Sensitivity of the maximum indentation depth to yield strength. (c) Sensitivity of the maximum indentation depth to the friction coefficient; Figure S3: Interfacial states and microstructural characteristics of Al/W energetic composites. (a–c) SEM images and corresponding elemental mapping of W particles that were only embedded on the Al surface. (d–f) SEM images and corresponding elemental mapping of W particles fully embedded within the Al matrix. (g–i) TEM images and corresponding elemental mapping of W particles embedded on the surface and within the Al matrix; Figure S4: Microstructural morphology and elemental distribution of Al/W composites prepared at other collision velocities. (a–c) At a collision velocity of 300 m/s, the W particles bounced off the Al particles, leaving permanent indentations on the Al matrix. (d) SEM image of the particle in (a) after FIB milling. (e–g) SEM images of the Al/W composite fabricated at a collision velocity of ~700 m/s. (h,i) SEM images of the Al/W composite fabricated at a collision velocity of ~900 m/s; Table S1: Material properties used in finite element models; Table S2: Statistics on the embedded depth of W particles in the 1/4 region in Figure 5i.
