# Supplementary material for: Integrated Finite Element Modeling and High-Speed Impact Synthesis: A Novel Pathway for Embedded Al/W Energetic Composites
Source: Materials (Basel). 2026 Aug 1;19(15):3252. doi: 10.3390/ma19153252 (PMC13466890; doi:10.3390/ma19153252)
Supplement: Supplementary file 1 [file materials-19-03252-s001.zip › materials-4422418-supplementary.pdf]

Supporting Information

Integrated Finite Element Modeling and High-Speed Impact Synthesis: A Novel Pathway for Embedded Al/W Energetic Composites

Kunkun Song [1], Xin Yu [1] , Yi Lan [1] , Xiansheng Wang [1,*] and Gang Liu [1,*]

[1]*High Speed Aerodynamics Institute, China Aerodynamics Research and Development Center. Mianyang, 621000，P. R. China.*

* Corresponding author.

E-mail: xiswhat@163.com (X.W.); san0004@126.com (G.L.)

Table S1 Material properties used in finite element models

Parameter/material	Al	W
Density ($g.cm^{-3}$)	2710	19300
Young’s modulus (GPa)	70	405
Shear modulus (GPa)	26	161
Poisson’s ratio	0.35	0.28
Heat capacity ($J. kg^{-1}. K^{-1}$)	904	134
Melting temperature (K)	933	3720
A (MPa)	148.4	1220
B (MPa)	345.5	1030
n	0.183	0.060
C	0.001	0.034
m	0.895	0.4
Reference temperature (K)	293	298
Reference strain rate (s^{-1})	1	1

Table S2 Statistics on the embedded depth of W particles in the 1/4 region in Figure 5i

Number	Embedded depth (μm)	Number	Embedded depth (μm)	Number	Embedded depth (μm)
1	0.117	22	1.530	43	1.789
2	0.259	23	1.685	44	1.802
3	0.557	24	0.596	45	1.970
4	0.739	25	1.063	46	2.048
5	0.920	26	1.478	47	2.346
6	1.089	27	2.048	48	0.920
7	1.335	28	0.441	49	1.594
8	1.465	29	1.102	50	1.685
9	1.594	30	1.206	51	2.178
10	1.737	31	1.478	52	2.657
11	1.763	32	1.659	53	2.385
12	1.607	33	1.802	54	1.193
13	1.335	34	2.100	55	1.504
14	1.867	35	2.061	56	2.009
15	0.402	36	2.541	57	2.333
16	1.685	37	1.037	58	2.981
17	1.944	38	0.739	59	2.852
18	0.389	39	1.063	60	4.083
19	0.907	40	1.206	61	5.367
20	1.556	41	1.231	62	6.974
21	3.552	42	1.426		
**Average embedding depth**				1.7255	

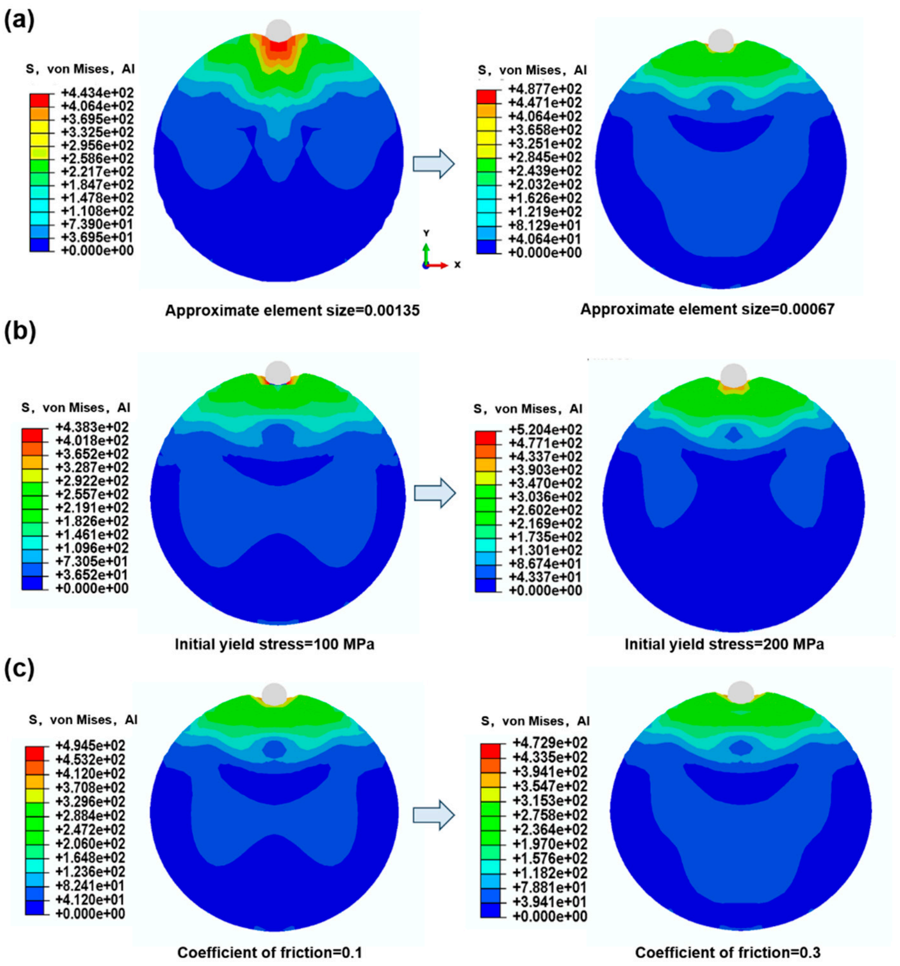

Figure S1 Sensitivity analysis of the effects of mesh size, the key J-C model parameters, and contact conditions on stress contours. (**a**) Influence of mesh size. (**b**) Influence of yield strength. (**c**) Influence of friction coefficient.

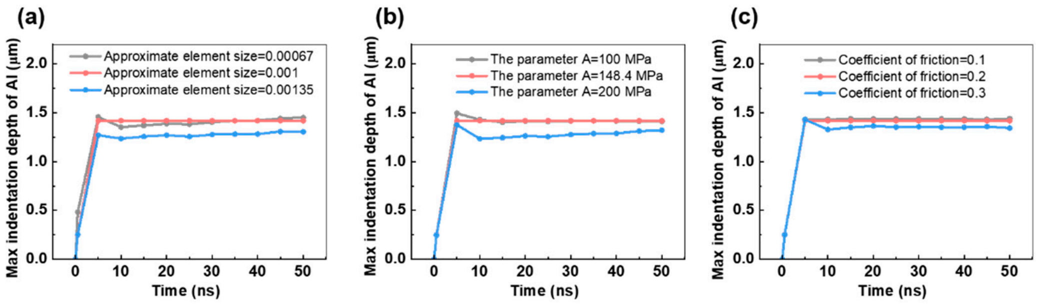

Figure S2 Sensitivity of the maximum indentation depth in the Al matrix to variations in mesh size, the key parameters of the J-C model, and contact conditions. (**a**) Sensitivity of the maximum indentation depth to mesh size. (**b**) Sensitivity of the maximum indentation depth to yield strength. (**c**) Sensitivity of the maximum indentation depth to the friction coefficient.

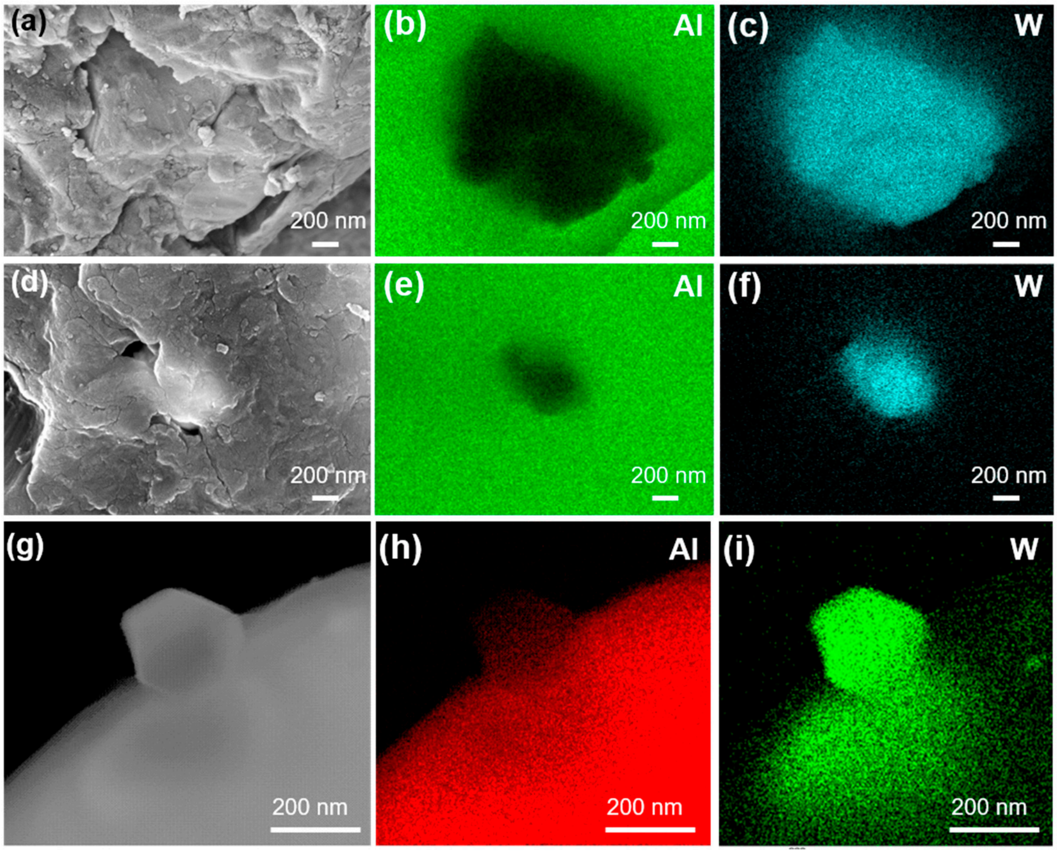

Figure S3 Interfacial states and microstructural characteristics of Al/W energetic composites. (**a**–**c**) SEM images and corresponding elemental mapping of W particles that were only embedded on the Al surface. (**d**–**f**) SEM images and corresponding elemental mapping of W particles fully embedded within the Al matrix. (**g–i**) TEM images and corresponding elemental mapping of W particles embedded on the surface and within the Al matrix.

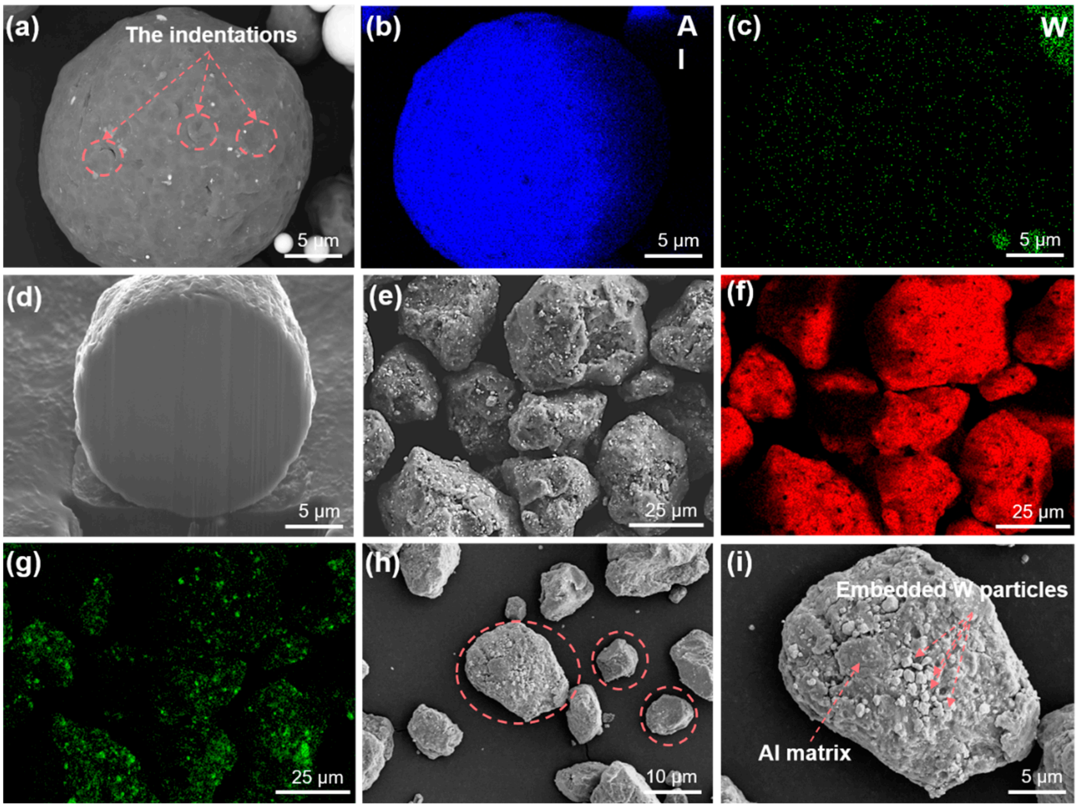

Figure S4 Microstructural morphology and elemental distribution of Al/W composites prepared at other collision velocities. (**a**–**c**) At a collision velocity of 300 m/s, the W particles bounced off the Al particles, leaving permanent indentations on the Al matrix. (d) SEM image of the particle in (a) after FIB milling. (**e**–**g**) SEM images of the Al/W composite fabricated at a collision velocity of ~700 m/s. (**h**,**i**) SEM images of the Al/W composite fabricated at a collision velocity of ~ 900 m/s.
